# Needs, gaps and opportunities for standard and e-mental health care among at-risk populations in the Asia Pacific in the context of COVID-19: a rapid scoping review

**DOI:** 10.1186/s12939-021-01484-5

**Published:** 2021-07-12

**Authors:** Jill K. Murphy, Amna Khan, Qiumeng Sun, Harry Minas, Simon Hatcher, Chee H. Ng, Mellissa Withers, Andrew Greenshaw, Erin E. Michalak, Promit Ananyo Chakraborty, Karen Sharmini Sandanasamy, Nurashikin Ibrahim, Arun Ravindran, Jun Chen, Vu Cong Nguyen, Raymond W. Lam

**Affiliations:** 1 Department of Psychiatry, Faculty of Medicine; APEC Digital Hub for Mental Health, University of British Columbia, 2255 Wesbrook Mall, Vancouver, BC V6T 2A1 Canada; 2 Department of Psychiatry, Faculty of Medicine, University of British Columbia, 2255 Westbrook Mall, Vancouver, BC V6T 2A1 Canada; 3 Sol Price School of Public Policy, University of Southern California, 650 Childs Way, Los Angeles, CA 90089 USA; 4 Global and Cultural Mental Health, Level 4, 207 Bouverie Street, Melbourne, Australia; 5 The University of Melbourne, Melbourne School of Population and Global Health, Carlton, Victoria 3010 Australia; 6 Department of Psychiatry, Faculty of Medicine, University of Ottawa, Roger Guindon Hall, 451 Smyth Road, Ottawa, ON K1H 8M5 Canada; 7 Healthscope Chair of Psychiatry, Department of Psychiatry, The University of Melbourne, Carlton, Victoria 3010 Australia; 8 USC Keck School of Medicine, USC Institute on Inequalities in Global Health, Los Angeles, USA; 9 APRU Global Health Program, 2001 N Soto Street SSB 318G, Los Angeles, CA 90089 USA; 10 Department of Psychiatry, Scientific Director, APEC Digital Hub for Mental Health, Faculty of Medicine & Dentistry, 4-142M Katz Group Centre for Pharmacy and Health Research, University of Alberta, Edmonton, Alberta T6G 2B7 Canada; 11 APEC Digital Hub for Mental Health, University of British Columbia, 420-5950 University Boulevard, Vancouver, BC V6T 1Z3 Canada; 12 School of Population and Public Health, University of British Columbia, 2206 East Mall, Vancouver, BC V6T 1Z3 Canada; 13 Mental Health, Substance Abuse and Violence Injury Prevention, Non-Communicable Disease Section, Disease Control Division, Ministry of Health, Level 2, Block E3, Putrajaya, Malaysia; 14 Precinct 1, Federal Government Administrative Complex, 62590 Putrajaya, Malaysia; 15 Global Mental Health Affairs & The Office of Fellowship Training, Department of Psychiatry, Graduate Faculty, Institute of Medical Sciences, University of Toronto, Toronto, Canada; 16 Campbell Family Mental Health Research Institute, Centre for Addiction and Mental Health, 100 Stokes St, Toronto, ON M6J 1H4 Canada; 17 Office for Clinical Research Center, Shanghai Mental Health Center, Shanghai Jiao Tong University School of Medicine, 600 South Wan Ping Rd, Xuhui District, Shanghai, China; 18 Institute of Population, Health and Development, ICON4 Tower, 243a Đường La Thành, Láng Thượng, Đống Đa, Hà Nội, 117222 Vietnam; 19 Department of Psychiatry, University of British Columbia, Vancouver, Canada; 20 Mood Disorders Centre, Djavad Mowafaghian Centre for Brain Health, Vancouver, Canada; 21 APEC Digital Hub for Mental Health, 2255 Wesbrook Mall, Vancouver, BC V6T 2A1 Canada

**Keywords:** COVID-19, Mental health, Equity, Asia Pacific, E-mental health, At-risk populations

## Abstract

**Background:**

The COVID-19 pandemic is expected to have profound mental health impact, including in the Asia Pacific Economic Cooperation (APEC) region. Some populations might be at higher risk of experiencing negative mental health impacts and may encounter increased barriers to accessing mental health care. The pandemic and related restrictions have led to changes in care delivery, including a rapid shift to the use of e-mental health and digital technologies. It is therefore essential to consider needs and opportunities for equitable mental health care delivery to the most at-risk populations. This rapid scoping review: 1) identifies populations in the APEC region that are at higher risk of the negative mental health impacts of COVID-19, 2) identifies needs and gaps in access to standard and e-mental health care among these populations, and 3) explores the potential of e-mental health to address these needs.

**Methods:**

We conducted a rapid scoping review following the PRISMA Extension for Scoping Reviews (PRISMA-ScR). We searched Medline, Embase and PsychInfo databases and Google Scholar using a search strategy developed in consultation with a biomedical librarian. We included records related to mental health or psychosocial risk factors and COVID-19 among at-risk groups; that referred to one or more APEC member economies or had a global, thus generalizable, scope; English language papers, and papers with full text available.

**Results:**

A total of 132 records published between December 2019 and August 2020 were included in the final analysis. Several priority at-risk populations, risk factors, challenges and recommendations for standard and e-mental health care were identified. Results demonstrate that e-mental health care can be a viable option for care delivery but that specific accessibility and acceptability considerations must be considered. Options for in-person, hybrid or “low-tech” care must also remain available.

**Conclusions:**

The COVID-19 pandemic has highlighted the urgent need for equitable standard and e-mental health care. It has also highlighted the persistent social and structural inequities that contribute to poor mental health. The APEC region is vast and diverse; findings from the region can guide policy and practice in the delivery of equitable mental health care in the region and beyond.

## Introduction

The novel coronavirus (COVID-19) pandemic has had unprecedented and devastating effects globally, including throughout the Asia Pacific region. The need for enhanced and targeted mental health care has been identified as urgent from the beginning of the outbreak in late 2019 [1]. Emerging research suggests there has been a global increase in common mental disorders during the pandemic [2], and the full extent of the mental health impact of COVID-19 will become increasingly apparent as the longer-term impacts of social isolation, job and economic insecurity, experiences of illness and bereavement, physical distancing, and disrupted access to usual health and mental healthcare reverberate among populations [3]. While the effects of COVID-19 are felt globally, some subpopulations, including those who have historically been marginalized and those on the front lines, may be particularly vulnerable [4]. In the context of the pandemic, the interplay of vulnerabilities such as existing mental illness and ongoing experiences of marginalization may increase the risk of negative mental health effects and exacerbate barriers to care [5] It is therefore essential to understand the needs of these populations and the barriers they may face in order to identify specific strategies to promote equitable access to mental health services by priority at-risk populations.

The COVID-19 pandemic has had implications for the delivery of both standard and virtual mental health care and has contributed to what constitutes a rapid “paradigm shift” in many aspects of society. Healthcare service delivery has, in many cases, shifted toward online, virtual, and tele-health models to maintain physical distancing during the pandemic [6, 7]. The spectrum of these types of interventions, referred to in this paper as ‘e-mental health or digital technologies’, include mental health care and psychosocial supports offered via telephone, video conferencing, text messaging, online tracking, education and management programs including Smartphone applications (apps) and other types of care or supports delivered via telephone or Internet technologies [7].

There is recognition of the great potential of e-mental health technology to address gaps in access to mental health care. In the context of COVID-19, there has been a call to increase the use of e-mental health care [8] and to prioritize it in the COVID-19 mental health research agenda [4]. Though e-mental health may improve access in high and low and middle-income countries (LMICs) [9–10], there are also risks that it might exacerbate inequities in access to care among high-risk populations who may experience low access to digital resources or other barriers [7, 11, 11]. The challenge of ensuring equitable access to e-health care in general among historically marginalized groups in the context of the pandemic has been recognized [12]. When exploring e-mental health options available in Canada during the pandemic however, a recent review noted that little assessment of equity issues exists in the literature [12]. Though more attention to this issue is emerging [13], comprehensive international evidence regarding equitable access to mental health care by specific at-risk populations is limited thus far. This demonstrates the need for equity-oriented research, recognizing the intersections of sex, gender, age, ethnicity and other factors, to identify needs and gaps to equitable mental health care delivery, including both standard and e-mental healthcare approaches [14, 15].

**Fig. 1:**
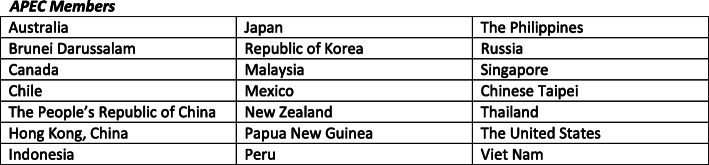
APEC Members

This paper is part of a larger study (Technology and Equitable Access for Mental Health Care in a post-COVID Asia Pacific- TEAM-CAP) examining the needs, challenges and opportunities related to e-mental health care in the Asia Pacific Economic Cooperation (APEC) region, bringing together policy makers, service providers and people with lived experience across the region. APEC is a regional economic forum with the objective to “create greater prosperity for the people of the region by promoting balanced, inclusive, sustainable, innovative and secure growth and by accelerating regional economic integration.” APEC is made up of 21 member economies (Fig. 1), referred to in this paper as “members”. This study is conducted by the APEC Digital Hub for Mental Health (‘the Digital Hub’), which acts as the coordinating centre for APEC mental health initiatives [16]. This review focuses on APEC members, which represent a diversity of experiences, challenges, and approaches to managing the pandemic. Members of APEC have taken several approaches to the development and rollout of standard and e-mental health care. This collective experience offers important opportunities for cross-regional learning and knowledge mobilization of equity-based considerations to guide policy and practice for standard and e-mental healthcare delivery in the context of COVID-19 and beyond.

This rapid scoping review has the following objectives: 1) to identify priority populations in the APEC region that are at higher risk of the negative mental health impacts of COVID-19, 2) to understand needs and gaps in access to standard and e-mental health care among these populations, and 3) to explore the potential of e-mental health to address these needs in vulnerable populations.

This study advances research on health equity in the context of the COVID-19 pandemic in several ways. First, it examines risk factors, needs and gaps across several priority at-risk population groups as identified in the literature. It also comprehensively captures emerging evidence from a diverse region that includes high, middle- and low-income members and which represents a variety of experiences and approaches to managing the pandemic and its impact on mental health. Finally, it focuses on equity related to e-mental health, an under-explored area, the urgency of which is gaining increased recognition in the context of the psychosocial impacts of the COVID-19 pandemic worldwide. At the time of writing, the COVID-19 pandemic is still ongoing, with research on the mental health impact continually emerging. This review provides an overview of findings from the early stages of the pandemic and advances considerations to inform equity-oriented mental health policy and practice through the remainder of the pandemic and beyond.

## Methods

The nature of our research question and the emerging evidence during an unprecedented global health crisis meant that a scoping review was warranted [17]. In recognition of the need for timely knowledge generation during the pandemic, our scoping review methods were informed by guidelines for conducting rapid reviews, allowing a streamlined approach to review methodology [18]. We describe our methodology below following the PRISMA Extension for Scoping Reviews Checklist [19].

Given the rapid nature of this review, a protocol has not been registered. This rapid scoping review combines the results of two searches. The first review was conducted from June 4th–12th 2020 using Medline and Google Scholar in addition to snowballing from reference lists. Search terms included Mental Health AND At-Risk Groups / Vulnerable Populations AND COVID-19 AND Asia-Pacific. Eligibility criteria were peer-reviewed papers published from December 2019 until June 1st 2020, related to mental health or psychosocial risk factors and COVID-19 among at-risk groups; that referred to one or more APEC members or had a global, thus generalizable, scope; English language papers, and papers with full text available. We excluded records that were unrelated to mental health, that described the general population instead of at-risk priority populations, were focused on countries outside of the APEC region, or were not in English. Thirty-six records were included in the initial review.

We conducted an updated and more expansive search of Medline, Embase and PsychInfo databases between August 8–10, 2020 for records published between January 1st and July 31st 2020. Working with a medical research librarian at the University of British Columbia, we developed a list of expanded search terms based on the results of the initial review. Eligibility criteria included records that described COVID-19; mental health; at-risk groups; and countries in the Asia-Pacific region. A full list of search terms is provided in Appendix A. Inclusion and exclusion criteria were as described above. Our search terms included several specific at-risk groups based on the results of our initial review, in addition to general terms including “vulnerable populations” and “at-risk” groups, allowing us to identify records related to populations previously identified in the literature as at-risk, while also enabling the emergence of previously unidentified population groups.

Data charting was conducted using a data extraction framework form with the following categories: article type, country/region, research question, study population(s), interventions, risk factors, needs and gaps for standard and e-mental health care, study conclusions and recommendations. Data extraction was conducted by JKM, AK and QS. Based on the data extracted into the framework forms, lead author JKM synthesized the data according to at-risk population, focusing on key themes emerging from the literature.

## Results

A total of 321 records were identified and 283 were eligible for the screening process after removal of duplicates. Title and abstract review were conducted by JKM and AK. After reviewing abstracts, 136 records were identified for full-text review. Full text review was conducted by JKM, AK and QS, after which a total of 95  records met our inclusion criteria and were included. This review includes these 95 records, in addition to the 36 included in the initial rapid review, for a total of 131  records. Given the diversity of the included papers and the emergent nature of the literature, a critical appraisal was not conducted. To capture a diversity of perspectives in this emergent field, we included a broad scope of published papers including primary research studies [20] and non-research-based papers including commentaries [21], letters and correspondence [20], reviews [13], perspectives and viewpoints [1], editorials [1], recommendations [2], clinical observations and notes from the field [3], and brief reports [22]. Table 22 lists type of paper, population(s) and country (ies) of focus for each included record.

**Table 1:** List of Articles Included in the Rapid Review Analysis

Reference	Type of Article	Population Type	Country or Region
Alavi et al. (2020) [23]	Primary research - modifiedDelphi methodology	People living with MNS disorders	United States
Albott et al. (2020) [24]	Review	Healthcare workers	United States
Baloran (2020) [25]	Cross-sectional study	Children and youth	Philippines
Baptiste et al. (2020) [26]	Editorial	Black, Indigenous and People of Colour	United States
Becker and Gregory (2020) [27]	Editorial	Children and youth	Global
Benhamou and Piedra (2020) [28]	Recommendations	Healthcare workers	United States
Bojdani et al. (2020) [29]	Review	People living with MNS disorders	United States
Boyraz and Legros (2020) [30]	Review	Multiple populations	Global
Brown and Weissman (2020) [31]	Letter	Older adults/people living with HIV	United States/Global
Buenaventura et al. (2020) [32]	Commentary	Older adults	Philippines
C. Liu et al. (2020) [33]	Cross-sectional study	Healthcare workers	China
C.K.T. Lima et al. (2020) [34]	Letter	Older adults	China
Campbell (2020) [35]	Review	Victims of domestic violence	Australia/Canada/United States
Cao et al. (2020) [36]	Cross-sectional study	Children and youth	China
Caqueo-Urízar et al. (2020) [37]	Commentary	Multiple populations	Chile
Chen et al. (2020) [38]	Correspondence	Healthcare workers	China
Courtenay and Perera (2020) [39]	Perspective	People with disabilities, chronic or pre-existing conditions	Global
Cui et al. (2020) [40]	Cross-sectional study	Children and youth/people living with MNS disorders	China
D. Liu et al. (2020) [41]	Cross-sectional study	COVID-19 patients/healthcare workers/patients with low socioeconomic status	Wuhan, China
D. Yang et al. (2020) [42]	Cross-sectional study	Children and youth	Wuhan, China
Davis et al. (2020) [43]	Perspective	Children and youth/people living with MNS disorders	Singapore
De Sousa et al. (2020) [44]	Review	Multiple populations	Low- and middle-income countries (LMIC)
Dell et al. (2020) [45]	Letter	Older adults/people with existing MNS disorders	Global
DeLuca et al. (2020) [46]	Review	Children and youth/people living with MNS disorders	United States
Druss (2020) [20]	Viewpoint	People living with MNS disorders	United States
Duan et al. (2020) [47]	Cross-sectional study	Children and youth	China
Duane et al. (2020) [48]	Commentary	Black, Indigenous and People of Colour	United States
Dumas et al. (2020) [21]	Primary research - online survey	Children and youth/people with existing MNS disorders	Canada
Efuribe et al. (2020) [49]	Commentary	Children and youth	United States
Emezue (2020) [50]	Viewpoint	Victims of domestic violence	Global
Rashidi Fakari and Simbar [51]	Letter	Pregnant and post-partum people	Global
Fernandez-Aranda et al. (2020) [52]	Editorial	People living with MNS disorders	Global
Fish et al. (2020) [53]	Primary research	Children and youth/LGBTQ+	United States
Fitzpatrick et al. (2020) [54]	Cross-sectional study	People living with MNS disorders	United States
Fortuna et al. (2020) [55]	Commentary	Black, Indigenous and People of Colour	United States
Furlong and Finnie (2020) [56]	Perspective	Black, Indigenous and People of Colour	Australia
Gao et al. (2020) [57]	Cross-sectional study	People living with MNS disorders	China
Golberstein et al. [58]	Viewpoint	Children and youth	United States
Gordon and Borja (2020) [59]	Commentary	General population	Global
Gunnell et al. (2020) [60]	Commentary	People living with MNS disorders	Global
Hamm et al. (2020) [61]	Primary research – semi-structured qualitative interview	Older adults/people with existing MNS disorders	United States
Han et al. (2020) [62]	Cross-sectional study	Healthcare workers	China
Hao et al. (2020) [63]	Cross-sectional study	People living with MNS disorders	China (Southwest)
Hayden and Parkin (2020) [64]	Review	Healthcare workers	Global
Hewson et al. (2020) [65]	Commentary	Incarcerated populations	Global
Horesh and Brown (2020) [66]	Review/recommendations	People living with MNS disorders	Global
Hou et al. (2020) [67]	Letter	Children and youth	China
Hu et al. (2020) [68]	Cross-sectional study	Healthcare workers	China
Ijadi-Maghsoodi et al. (2020) [69]	Commentary	Children and youth	United States
J. Liu et al. (2020) [70]	Cross-sectional study	Children and youth	China
Jorm (2020) [71]	Editorial	People living with MNS disorders	Australia
Junior et al. (2020) [72]	Letter	Black, Indigenous and People of Colour	Global
Junior et al. (2020) [73]	Letter	Refugees and migrants	Global
Kang et al. (2020) [74]	Correspondence	Healthcare workers	China
Kannarkat et al. (2020) [6]	Viewpoint	People living with MNS disorders	United States
Kanzler and Ogbeide (2020) [75]	Commentary	Healthcare workers/people with existing MNS disorders	United States
Karamouzian et al. (2020) [76]	Commentary	People living with MNS disorders	Global
Kaufman et al. (2020) [77]	Editorial	People living with MNS disorders	Global
Kaukinen (2020) [78]	Review	Victims of domestic violence	Global
Kavoor (2020) [79]	Letter	People living with MNS disorders	Global
Khusid et al. (2020) [80]	Cross-sectional study	Healthcare workers	United States
Kim and Su (2020) [81]	Viewpoint	People living with MNS disorders	Global
Klomek (2020) [82]	Correspondence	People living with MNS disorders	Global
Ko and Yen (2020) [83]	Commentary	Children and youth/people living with MNS disorders	Global
L. Yang et al. (2020) [84]	Cross-sectional study	People living with MNS disorders	China
LaHue et al. (2020) [85]	Letter	Older adults	United States
Lai et al. (2020) [86]	Cross-sectional study	Healthcare workers	China
Langmaid et al. (2020) [87]	Clinical observations	People with disabilities, chronic or pre-existing conditions	United States
Diaz de Leon Martinez et al. [88]	Review	Black, Indigenous and People of Colour	Mexico
Li and Zhang (2020) [89]	Commentary	People living with MNS disorders	China
Liang et al. (2020) [90]	Cross-sectional study	Children and youth	China
Liem et al. (2020) [91]	Commentary	Refugees and migrants	Global
Lim et al. (2020) [92]	Cross-sectional study	Healthcare workers	Canada
Lin et al. (2020) [93]	Letter	Healthcare workers	Hubei, China
Liu and Modir (2020) [94]	Editorial	Black, Indigenous and People of Colour	United States
M.H. Li et al. (2020) [95]	Letter	Children and youth	Hong Kong
McGee et al. (2020) [96]	Recommendations	People with disabilities, chronic or pre-existing conditions	United States
Misra et al. (2020) [97]	Commentary	Black, Indigenous and People of Colour	United States
N.N.R. Lima et al. (2020) [98]	Letter	People experiencing homelessness	Global
Nie et al. (2020) [99]	Cross-sectional study	COVID-19 patients	Wuhan, China
Novacek et al. (2020) [100]	Recommendations	Black, Indigenous and People of Colour	United States
Pachana et al. (2020) [101]	Commentary	Older adults	Australia
Pozza et al. (2020) [102]	Perspective	People living with MNS disorders	Global
Price (2020) [103]	Cross-sectional study	People living with MNS disorders	Canada
Prime et al. (2020) [104]	Review	Children and youth	Global
Rodgers et al. (2020) [105]	Commentary	People living with MNS disorders	Global
Rothstein and Olympia (2020) [106]	Review/recommendation	Children and youth	United States
S. Yang et al. (2020) [107]	Cross-sectional study	Healthcare workers	South Korea
Santos et al. (2020) [108]	Cross-sectional study	People living with HIV	Global
Shakespeare-Finch et al. (2020) [109]	Commentary	Multiple populations	Australia
Shervington and Richardson (2020) [110]	Editorial	BIPOC populations	United States
Shiau et al. (2020) [111]	Commentary	People living with HIV	United States
Shigemura et al. (2020) [112]	Letter	Multiple populations	Japan
Singh (2020) [113]	Correspondence	People living with MNS disorders	Global
Sneed et al. (2020) [114]	Commentary	Black, Indigenous and People of Colour	United States
Song et al. (2020) [115]	Cross-sectional study	Healthcare workers	China
Stewart et al. (2020) [116]	Feasibility study	Children and youth	United States
Sun et al. (2020) [117]	Notes from the field	People living with HIV	China
Suzuki (2020) [118]	Primary research - cohort study	Pregnant and post-partum people	Japan
Talevi et al. (2020) [119]	Review	Multiple populations	Global
Tang et al. (2020) [120]	Cross-sectional study	Children and youth	China
Taylor et al. (2020) [121]	Commentary	People living with MNS disorders	United States
Thompkins et al. (2020) [122]	Commentary	Black, Indigenous and People of Colour	United States
Thomson et al. (2020) [123]	Review	People living with MNS disorders	Australia
Tracy et al. (2020) [124]	Editorial	Healthcare workers	Global
Tsai and Wilson (2020) [125]	Commentary	People experiencing homelessness	United States/Canada
Tu et al. (2020) [126]	Cross-sectional study	Healthcare workers	Wuhan, China
Usher et al. (2020) [127]	Editorial	Victims of domestic violence	Australia
Van et al. (2020) [128]	Cross-sectional study	People living with MNS disorders	Australia
Mesa Viera et al. [129]	Brief report	Multiple populations	Global
Viswanathan et al. (2020) [130]	Perspective	Healthcare workers	United States
W. Li et al. (2020) [131]	Primary research - cohort study	Healthcare workers	China
W. Zhang et al. (2020) [132]	Cross-sectional study	Healthcare workers	China
Wang et al. (2020) [133]	Correspondence	Children and youth	China
Wood (2020) [134]	Letter	People experiencing homelessness	Global
Wu and Wei (2020) [135]	Cross-sectional study	Healthcare workers	China
Wu et al. (2020) [136]	Cross-sectional study	Pregnant and post-partum people	China
Xiang et al. (2020) [1]	Commentary	Healthcare workers	China
Xiao et al. (2020) [137]	Cross-sectional study	Children and youth	China
Xie et al. (2020) [138]	Cross-sectional study	Children and youth	China
Xin et al. (2020) [139]	Cross-sectional study	Children and youth	China
Xing et al. (2020) [140]	Cross-sectional study	Healthcare workers	China
Xu et al. (2020) [141]	Letter	Healthcare workers	Shanghai, China
Y. Zhang et al. (2020) [142]	Longitudinal study	Children and youth	China
Yang et al. (2020) [143]	Correspondence	Older adults	China
Yao et al. (2020) [144]	Correspondence	People living with MNS disorders	China
Yin et al. (2020) [145]	Cross-sectional study	Healthcare workers	China
Yue et al. (2020) [146]	Cross-sectional study	Pregnant and post-partum people	China
Zhai and Du (2020) [147]	Correspondence	Children and youth	China
Zhu et al. (2020) [148]	Cross-sectional study	Healthcare workers	China

Table 2 lists the APEC member or region of focus of each study, with a majority originating from China, the United States or with a global focus. Where papers describe more than one APEC member specifically they are listed under each member (e.g. data from the US and Canada [125]).

**Table 2:** List of APEC members represented in the rapid review analysis

Member	Number of studies
Australia	8
Canada	5
Chile	1
The People’s Republic of China	42
Global	33
Hong Kong, China	1
Japan	2
Mexico	1
Philippines	2
Singapore	1
Republic of Korea	1
United States	35

Table 3 lists the types of populations described in the included studies and the types of studies identified. Some studies identified populations that are classified in more than one at-risk category (e.g. older adults living with HIV [31] and are therefore counted in both categories. Studies describing considerations for several different vulnerable populations are listed under “multiple populations”.

**Table 3:** List of at-risk populations described in the rapid review analysis

At-Risk Population	Total papers	Primary research papers (citations)	Non-research-based papers (citations)
People living with MNS* disorders	34	9 [21, 23, 54, 57, 61, 63, 84, 103, 128]	25 [6, 20, 29, 45, 46, 52, 59, 60, 66, 71, 75–77, 79, 81–83, 89, 102, 105, 112, 113, 121, 123, 144]
Healthcare workers	29	18 [33, 62, 68, 80, 86, 92, 93, 107, 115, 126, 131, 132, 135, 140, 141, 145, 148, 149]	11 [1, 24, 28, 38, 64, 74, 75, 112, 124, 129, 130]
Children and youth	28	16 [21, 25, 36, 40, 42, 47, 53, 67, 70, 90, 116, 120, 137–139, 142]	12 [27, 43, 46, 49, 69, 83, 95, 58, 104, 106, 133, 147]
Black, Indigenous and People of Color	14	2 [54, 122]	12 [26, 30, 48, 55, 56, 72, 88, 94, 97, 100, 110, 114]
Multiple populations	8		
Older adults	7	1 [61]	6 [31, 32, 34, 85, 101, 143]
COVID-19 patients	6	2 [41, 99]	4 [30, 44, 112, 119]
People with disabilities, chronic or pre-existing conditions	5	0	5 [30, 39, 87, 96, 129]
Refugees and migrants	5	0	5 [37, 73, 91, 109, 129]
People living with HIV	4	1 [108]	3 [31, 111, 117]
People experiencing domestic violence	4	0	4 [35, 50, 78, 127]
Pregnant and post-partum people	4	3 [118, 136, 146]	1 [51]
People experiencing homelessness	3	0	3 [98, 125, 134]
Incarcerated populations	2	0	2 [65, 129]

*MNS mental, neurological and substance use disorders

Results are described below, identifying mental health risk factors by population, followed by recommendations for standard and e-mental health care and psychosocial support for at-risk populations.

### Risk factors by at-risk population

#### People with existing mental, neurological and substance use (MNS) disorders

Papers on people living with existing MNS disorders (Table 3) cover a diverse spectrum of MNS conditions, including common mental disorders like major depressive disorder (MDD) [57, 6], Post Traumatic Stress Disorder (PTSD) [20], eating disorders [21, 21, 23], obsessive-compulsive disorder (OCD) [23], severe mental illness including schizophrenia and bipolar disorder [23, 23, 29, 43, 43, 45, 45, 46, 46, 52], substance use disorders [54, 59, 60], epilepsy [61], behavioural addictions (e.g. gambling [61], gaming disorder [63], chronic insomnia [66], intellectual and developmental disabilities (IDD) including autism [71], and suicide risk [75, 76, 77, 79], in addition to general considerations for mental health responses to COVID-19 [81, 82, 83, 83, 84, 89, 102, 103]. A majority of papers describe considerations for general adult populations, while some focus on children and youth [105, 113, 121, 123, 128] and older adults [144, 150].

Primary research studies report social and physical isolation as a risk factor for people living with MNS disorders during the pandemic. For example, cross-sectional studies in Canada found that risk of using substances alone among adolescents increased during social distancing measures and was associated with fears of COVID-19 and depressive symptoms [21], while another found an increase in online gambling and in predictive factors for problematic gambling among high-risk gamblers [103]. Physical isolation was also related to increased symptoms of anxiety and insomnia among people with pre-existing chronic insomnia in China [84], and worsening stress, depression, financial worry and an increase in adverse lifestyle behaviours among people living with mood disorders in Australia, particularly among men living with bipolar disorder [128]. A US study assessing potential suicide risk found that food insecurity, racialization, immigrant status, single people and families with children had increased risk of suicidality based on the revised Suicide Behaviours Questionnaire (SBQ-R) [54], although it is notable that studies have shown either no increase or a decline in suicide rates in high income countries in the earlier stages of the pandemic [151].

Interruption in usual care was also identified as a risk factor in primary research studies. A mixed methods study among older adults with pre-existing common mental disorders in the US found that disruptions in usual physical and mental healthcare were distressing [61], while a Chinese study including 570 outpatients with depression or anxiety reports that 70% of patients had to postpone their treatment due to pandemic-related restrictions [57]. Increased exposure to news and social media may also increase distress and exacerbate symptoms as suggested by results of a cross-sectional study assessing psychological distress among people living with epilepsy in China which found a significant association between time spent on media coverage of COVID-19 and severe psychological distress [63].

Other potential risk factors related to COVID-19 and people living with existing MNS disorders are raised in non-research-based papers. Patients with severe mental illness and/or substance use disorders may experience increased susceptibility to COVID-19 infection and related complications due to physical comorbidities, smoking, low socioeconomic status, poor housing conditions or housing instability, unemployment and social isolation [20, 29, 52, 57, 76, 77, 79]. People with existing MNS conditions might also have low health literacy, making it challenging for them to follow public health guidelines [81]. The impact of social isolation and other added stressors of the pandemic are also raised, including the potential contribution to worsening symptoms [6, 45, 46, 79, 81, 105, 123], other unhealthy behaviours [83] and suicidality [46, 60]. Stigma towards people with COVID-19 and MNS disorders might lead to ‘double stigma’, lowering help-seeking for both physical and mental health conditions and worsening mental health [45, 60, 77]. Patients living in inpatient and residential facilities face several risk factors, including high risk of COVID-19 transmission and severe social isolation due to limitations on family visits and group activities [79, 89]. For example, in a commentary describing risks facing psychiatric inpatients in China, Li et al. [89] note that electronic devices are not permitted for patients, leading to further isolation and psychological distress.

#### Healthcare workers

Health care workers (HCWs) also emerged from the literature as a priority at-risk population (see Table 3). Mental health risk factors identified by primary research studies among HCWs include high risk of exposure to COVID-19 via direct contact with patients [33, 62, 68, 93, 107, 145], insufficient availability of personal protective equipment (PPE) [68, 80, 86, 148, 149], overwork [86, 126, 140, 148], and feeling unsupported by superiors or management [1, 24]. In several studies in China, female gender was associated with higher levels of psychological distress among HCWs [24, 24, 28, 28] as was younger age [62, 62], though one study reported higher rates of depression and PTSD among male health workers [62]. Additional risk factors raised in non-research based papers included rapidly changing demands on HCWs [64], uncertainty related to the virus [64, 80], concerns about infecting family members [86, 86, 115, 129, 130], and financial worries [140, 145]. For HCWs in low and middle-income countries, limited resources may lead to higher risk of exposure to COVID-19 and added stress [148].

Specific types of providers experience different stressors. In primary research studies, nurses were found to be particularly vulnerable to the negative mental health impacts of the pandemic [62, 86], likely due to their increased and prolonged contact with COVID-19 patients compared with other HCWs. Physical therapists in South Korea reported fear of infection due to close proximity required by the nature of their profession [107], while radiologists working in Sichuan Province in China reported higher anxiety levels than the general population, but lower levels compared with other types of HCWs [149]. Nurses conscripted to work in Wuhan, China during the pandemic reported mental health risk factors including social isolation and being away from family, in addition to factors such as overwork and risk of infection [126]. Emergency room physicians in Canada surveyed immediately before the pandemic had high levels of burnout, leading to increased risk of depression, harmful substance use and suicidality [92], which the authors note is of great concern given the added pressures of the COVID-19 pandemic.

Several primary research studies reported increased symptoms of anxiety [33, 62, 68, 86, 93, 107, 126, 132, 141, 149]. For example, one cross-sectional study [86] identified prevalence of anxiety of 44.6% among 1257 healthcare workers in 34 hospitals treating COVID-19 patients in China, while another found that 40% of conscripted nurses in Wuhan, China reported symptoms of anxiety [126]. Among physiotherapists in South Korea, 32.3% reported anxiety symptoms. Depression symptoms among HCWs were also elevated [62, 68, 86, 92, 93, 24, 86, 86, 93, 107, 115]. A study of Chinese HCWs directly working with COVID-19 patients found depression rates of 50.4%, while emergency department staff in China [115] had depression rates of 25.2%. Studies also found elevated risk of post-traumatic stress syndrome (PTSS) and PTSD symptoms [126] and insomnia [126, 132, 132, 135, 135] among HCWs. HCWs may also experience subclinical symptoms that might also greatly impact their work functioning and quality of life [141]. For example in Lai et al.’s study of HCWs treating COVID-19 patients across China, 71.5% had symptoms of distress based on the Impact of Event Scale-Revised (IES-R) [145].

#### Children and youth

Children and youth were the third most highly-represented priority population identified in the literature (see Table 3). Primary research studies indicate that children and youth are already at high risk of poor mental health, with COVID-19 expected to worsen this risk [116]. In a cross-sectional survey of *n* = 3613 youth from 20 provinces in China, depression rates of 22.28% were found during the pandemic, compared with pre-pandemic rates of 13.2% [47]. Social distancing requirements may have a negative impact on youth mental health [36, 47, 137], in part due to separation from important social support networks [53, 70]. Restrictions on leaving the home may have a negative impact on lifestyle factors such as increased screen time, less exercise, and increased substance use [21, 137, 138]. The pandemic has also resulted in increased fear among children and youth, negatively impacting their mental health [40, 42, 47, 120, 138, 142]. Youth, such as *n* = 530 high school and college students surveyed in the Philippines, expressed worries about financial and food security related to the pandemic [25]. In non-research based papers, suggested risk factors include the effects of restrictions on excessive gaming [83], increased screen time [133], and poor sleep habits [27]. COVID-19 restrictions and economic impact might also place strain on parents and caregivers, increasing conflict at home [104, 106].

School closures also appear to have an impact on youth mental health [67]. Cross-sectional studies from China reported high mental health risk among students, with mental health risk factors among students including being in final year of study and living in harder hit regions or rural areas [47, 58] . An editorial from Hong Kong [69] suggests that students taking national exams are at higher risk of poor mental health. School closures may also mean that children and youth might be cut off from crucial mental health and other supports delivered at schools. This is especially true for youth who are racialized, from low socioeconomic backgrounds, and from families with no health insurance [95, 106, 120]. Contexts with existing and ongoing social and political unrest, including in Hong Kong where student-led protests had already led to restrictions on movement prior to the pandemic, may also contribute to mental health risk.

Vulnerable subpopulations among youth may be at high risk of negative mental health impacts. In a qualitative study analysing *n* = 31 chats on an online support service for LGBTQ+ youth in the US, being at home with unsupportive family members and being cut off from ‘safe spaces’ and communities was identified as a mental health risk factor [53]. In a correspondence piece, Zhai and Du [147] state that Chinese students living abroad early in the pandemic experienced fear related to the safety of their family members and experienced discrimination and stigma based on racist misconceptions about the pandemic.

#### Black, indigenous and people of colour (BIPOC) populations

BIPOC populations are also identified as a priority at-risk population (Table 3). BIPOC populations may face several mental health risk factors as a result of structural marginalization and discrimination in the APEC region, though perspectives from the literature identified in this review predominantly describe the US, with one paper each from Australia [56] and Mexico [88]. BIPOC communities may face an elevated health risk due to the effects of structural marginalization and the social determinants of health, putting them at higher risk of contracting COVID-19, of developing complications, and of experiencing negative mental health and psychosocial effects. For example, Indigenous Australians have a higher risk of NCDs, smoking, mental illness and risk of suicide compared with the general population [56], leading to a rapid targeted response to curb the spread of COVID-19 among Indigenous communities [26]. Black Americans are disproportionately represented among COVID-19 cases and deaths. In a commentary describing the situation in Michigan, where Black Americans make up 13% of the population, they represent 32% of cases and 41% of deaths related to COVID-19 [26]. In New Orleans, which emerged as an epicentre early in the pandemic, the disproportionate impact of COVID-19 was linked to persistent racial and socioeconomic inequity [30]. Black Americans have higher rates of cardiovascular disease and are more likely to live in densely populated areas [54]. Black and Latinx communities in the US also face structural racism that means that many neighbourhoods are underserved by hospitals, pharmacies and COVID-19 testing facilities [55]. Undocumented Latinx individuals might fear accessing testing or healthcare services due to possible immigration reprecussions [88]. A cross-sectional study in the US found higher rates of COVID-19 related fear among Asian, Latinx and foreign-born individuals, which in turn was correlated with elevated mental health symptoms [94]. In the US, Black, Latinx and Asian populations make up 70% of the essential workforce, increasing their risk of exposure [94]. These jobs often have limited or no paid sick leave, no health insurance coverage and may be precarious in times of crisis [94, 94, 110, 114]. A commentary reviewing risk factors for Indigenous communities in Mexico describes elevated rates of poverty and extreme poverty, as well as lower indicators for education, food security, housing and social security. Some Mexican Indigenous communities lack basic water, sanitation services and health services, which might put them at elevated risk of COVID-19 infection and psychosocial distress [152].

Access and utilization of mental health care by BIPOC populations may also be low [55]. Populations such as Black Americans [100, 114, 122] and Indigenous Australians [56] may lack trust in health and mental health services due to historic experiences of trauma and structural marginalization which might impact help-seeking. High rates of mental health related stigma [114], lack of insurance coverage and absence of culturally appropriate care [56, 114, 88] also act as barriers to mental healthcare access for BIPOC populations. Past histories of trauma and current trauma may exacerbate the mental health impact of the COVID-19 pandemic for BIPOC people. Witnessing the impact of COVID-19, including via extensive media coverage of the impact of the virus on Black people in the US [114] for example, is a mental health risk factor. Recent and persistent traumas also play a role, including the protests and unrest related to racial injustice in the US, recent bush fires in Australia [109] and forest fires in California, the water crisis in Flint, Michigan [114], and Hurricane Katrina [110, 122]. For communities with a history of trauma, measures such as stay-at-home orders may evoke mental distress related not only to COVID-19 but also to past traumatic events [122]. The disproportionate representation of BIPOC people among COVID-19 cases and deaths means that these communities are likely disproportionately facing fear and bereavement, putting them under substantial psychological strain [114] with interruptions in religious [122] and funeral services [114] impacting bereavement processes and access to community support.

An increase in xenophobia and racist incidents, including at the individual, structural and political level, is a risk factor for negative mental health effects of COVID-19 [56, 94, 97]. Increased anti-Asian stigma and discrimination during the COVID-19 pandemic in the US is described as a risk factor for poor mental health outcomes among Asia Americans, including anxiety, depression and general distress, especially when combined with other pandemic-related stressors [97]. Racial discrimination in healthcare delivery, including anticipation of discrimination by healthcare providers based on racialization, may be associated with increased depression, anxiety and post-traumatic stress disorders [114].

#### Older adults

Older adults also experience considerable mental health risk factors in the context of COVID-19. In China, which has the world’s highest population of people over 60 years, depression rates among older adults prior to COVID-19 were 23.6% [143], suggesting the added stressors of the pandemic may exacerbate symptoms. Older adults living with dementia and existing mental illnesses, including those living in long-term care homes, might experience worsening symptoms due to lack of family visitation and restrictions on social activities [101]. Mental health care access for older adults has been limited due to quarantine and physical distancing [34, 143]. Disruptions in mental health and other health services such as elective surgeries might also lead to increased stress and worry, as identified in a mixed methods study among older adults with common mental disorders in the US [61]. For hospitalized older adults, restrictions on interventions such as delirium prevention measures may lead to higher incidence of mental health and cognitive disturbance [85].

Social isolation and confinement as a result of the pandemic is a risk factor for poor mental health and cognitive decline [32, 101]. Many older people live alone [32], putting them at risk of loneliness and poor mental health [32], and confinement and fear about contracting COVID-19 might contribute to increased anxiety and depression [32, 61]. Disruption of community support and social activities further isolates from social connection and meaningful activities [101, 101]. Older adults might also fear dying alone or be unable to properly grieve loved ones due to pandemic restrictions [101]. Ageism has also been prominent during the COVID-19 pandemic, with stigma about older people and their place in society widely expressed [143].

Some subpopulations of older adults might also experience elevated risk factors. Older adults living in poverty face added challenges. For example, a commentary from the Philippines states that only 30% of older Filipinos receive a pension, meaning that many older adults are unable to pay healthcare costs and medical bills, especially in rural areas [32]. In Australia, deaths resulting from the severe bush fires immediately prior to the COVID-19 pandemic occurred disproportionately in people aged 60–69 years, meaning that older adults were already under enormous psychological strain. The added stressors of the COVID-19 pandemic have serious mental health implications [101]. Among older adults living with HIV, many already experience social isolation, loneliness and stigma. The COVID-19 pandemic may lead to interruptions in HIV and mental health treatment access, increased financial strain and double stigma which has additional mental health consequences [31].

#### Additional priority at-risk populations

Other priority at-risk populations are described in the literature, though the number of studies is lower than for the populations already discussed (see Table 3). For *patients with COVID-19,* mental health risk factors include challenges accessing hospital care, physical and social isolation, witnessing the death of other patients or the death of family members, and negative mental health effects related to COVID-19 treatment [41, 119]. A review based on early COVID-19 research and previous research from the SARS outbreak notes that COVID-19 patients are likely at higher risk of PTSD [30]. People diagnosed with COVID-19 may also experience loss of salary resulting in economic hardship [44]. A cross-sectional survey among COVID-19 patients discharged from hospital in Wuhan, China found that severe disease was a strong risk factor for PTSD and depression, while perceived risk of discrimination based on prior COVID-19 infection was a risk factor for PTSD, depression and anxiety [41]. In another cross-sectional study assessing rates of depression and anxiety among COVID-19 patients in Wuhan, 35.9% of patients had depression, 38.5% had anxiety and 24.3% had both, with risk for both depression and anxiety diagnosis higher among women [99].

*Incarcerated populations* are also identified as high-risk of negative mental health impact during the pandemic. They often live in overcrowded conditions, already have high rates of physical and mental illness, substance use disorders and suicidality and have poor access to health and mental healthcare [65, 65]. During the COVID-19 pandemic, many jury trials and court dates have been delayed, leading to longer remand time, with concomitant additional strain. Additionally, prison visits which can help support mental health have largely been suspended during the pandemic. Suspension of activities in prison to control the spread of COVID-19 can lead to long periods of time spent alone in cells [129], contributing to psychological distress.

*People living with HIV (PLWH)* are also identified as an at-risk population, and experience elevated rates of mental health and substance use disorders related to increased experiences of marginalization and stigma [111, 117]. Older PLWH already experience social isolation, which might increase during the pandemic leading to mental and cognitive decline and reduction of self-care practices [31, 108]. Many PLWH, including sexual minority and BIPOC people, might not be able to physically distance due to work or housing circumstances [108]. Interruptions in care and medication access for PLWH during the pandemic is also a risk factor [111, 111]. A cross-sectional survey among *n* = 703 PWLH in China found that 60.8% reported depression and 49.8% reported symptoms of anxiety. Disruptions in access to usual care can cause mental distress among PLWH. In China, hospitals and HIV clinics began mailing ART treatments during the pandemic, which caused distress related to the risk of disclosure to family members [117]. Practices such as contact tracing can also increase vulnerability for gay PLWH or men who have sex with men (MSM) due to risks associated with privacy violations, homophobia and discrimination [117].

*People who are pregnant or post-partum* are identified as at-risk as they may experience increased fear and anxiety related to COVID-19 [146], which can increase risk of pregnancy complications including antenatal depression [118, 51]. Stressors may include being forced to deviate from birth plans and give birth without family being present and limited physician visits due to fear of infection [51]. Cross-sectional studies in China found elevated rates of anxiety [146] and depression [136] among pregnant women during the COVID-19 pandemic.

*Refugees and migrants*, including migrant workers, might encounter language barriers and other challenges with access to information, struggle with sociocultural differences in receiving countries, including in healthcare settings, and face precarious housing conditions such as overcrowding and the impossibility of social distancing in refugee camps [73, 129]. In Chile, migrants, including asylum seekers, are stuck at the border, facing economic, health and psychosocial hardship [37]. In Australia, asylum seekers might experience precarious and overcrowded housing conditions and were excluded from financial aid packages, further contributing to their vulnerability [73]. International migrant workers, particularly domestic workers, face more barriers in accessing health care services compared to other migrants and have a higher burden of common mental disorders and lower quality of life compared with the general population [91]. The COVID-19 pandemic exacerbates this risk, due to factors such as lost income and isolation away from home countries. For example, many international migrant workers in Macau and Hong Kong face economic vulnerability as a result of the pandemic [91]. The high number of migrants and refugees in some settings, combined with interruptions in services because of the pandemic mean there are limited mental health and psychosocial support services available [109].

Risk factors for *people experiencing homelessness* include cramped living conditions and poor access to sanitation, limiting their ability to physically distance and engage in regular hygiene practices like handwashing [98]. People experiencing homelessness have high existing prevalence of mental health and substance use disorders and other comorbidities and limited support and outreach services during the pandemic [134]. Many people experiencing homelessness may be hard to reach through contact tracing due to being transient or fears of involuntary hospitalization or incarceration [125, 134].

For *people experiencing domestic or intimate partner violence*, the conditions of lockdown or physical distancing may compound risk factors for violence including isolation, economic strain, lack of access to support services and safe spaces and increase in alcohol consumption at home [35, 35, 78]. Evidence from post-disaster settings shows an increase in domestic violence following emergencies [78], and Australia saw an increase in domestic violence reports and Google searches related to support seeking early in the pandemic [127]. During the pandemic there may be reduced access to victim services, and lack of access to a secure place to call police, reach out for help or to research options for support [127].

Finally, *people with disabilities and/or chronic illness* often face persistent low access to care, and stigma, particularly in LMICs [129], and experience increased prevalence of mental health and physical comorbidities. Interruptions in usual care and routines may cause heightened distress and anxiety [30, 39]. Gaps in regular care, including in-home care workers, may also place a strain on the families and caregivers of people with disabilities and chronic disease, causing burnout and negative mental health impacts [39, 87]. People with intellectual disabilities such as autism might experience high levels of anxiety or exacerbation of OCD symptoms related to intense focus on COVID-19 related news, disruption in routine and need for enhanced handwashing [39]. People with conditions such as chronic respiratory diseases face elevated risk of COVID-19 infection and complications, and face mental health risk factors related to isolation and loneliness [96].

### Considerations for standard mental health care and psychosocial support

The need for government investment in targeted supports for at-risk groups is evident, with recommendations for investment to support populations such as HCWs [33, 36] and to invest in enhanced mental health support focussing on COVID-19 patients, for example [42]. Organizational support by various institutions is also called for. For example, studies from China on HCW mental health [62, 68] recommend targeted support programs for the most at-risk, such as nurses, women and those that are in direct contact with COVID-19 patients [86]. Studies note the important role of universities and colleges in providing support for vulnerable students including appropriate and timely mental health care for international students [92, 99, 120, 137, 147].

Increasing social support is also identified as critical. In China, for example, hospitals took steps to implement rotations and shifts that allowed HCWs time away from high-risk wards enabling them to spend time with family and friends [62]. For people with existing MNS disorders, enhanced support by community, family and friends was described as essential [60, 102, 113, 128]. Among people who have had COVID-19, efforts to reduce discrimination and increase social support were also recommended [41].

Some specific mental health interventions were recommended. For HCWs, recommended programs include cognitive behavioural therapy (CBT) [28] and peer support [24, 130], which focus on building resilience and self-efficacy and refocusing on a sense of professional purpose. Recommended interventions to support people living with MNS disorders include self-management strategies such as sleep hygiene, relaxation techniques, healthy behaviours in the context of COVID-19 (e.g. hand washing), self-efficacy and problem-solving [79, 84]. Based on a mixed methods study among older adults with existing MDD, psychoeducation was recommended for the general older adult population, including targeted messages on how to maintain safe social interactions and meaningful activities. For BIPOC populations, comprehensive and accessible programs that include early intervention [100, 110] and the delivery of culturally and linguistically competent, anti-racist and trauma-informed mental health care that focuses on strengths and resiliency are recommended [26, 72, 88, 94, 100, 114, 122].

An important aspect is the need to engage communities in the design, development and implementation of mental health and psychosocial supports that affect them. One example from China [38] was initial reluctance by HCWs to participate in psychosocial support programs offered by their hospital, but after consulting with HCWs, the hospital was able to implement organizational supports that directly responded to their needs and concerns, including provisions for them to live apart from their families and communicate via videoconferencing, opportunities for rest, and increased training. Among BIPOC populations, consulting with trusted community leaders and members to ensure that mental health care is appropriate and acceptable is recognized as essential [110, 114, 122], as is engaging youth directly in the development of programs and policies that support their mental health and well-being [49].

Ensuring continued access to the most appropriate care, including in-person care when needed, also emerged as an important consideration. For people with existing MNS disorders, in many contexts only people experiencing severe or urgent symptoms received in-person care. The lack of access to usual care presents a barrier for many people living with MNS disorders, as described in an editorial on challenges faced by people living with eating disorders, who might lack access to their usual care teams and find the increased need for self-management challenging [52]. Care for people with autism or severe intellectual disabilities is usually delivered in the community and with close physical contact. A needs assessment of mental health care services during the pandemic in the US found that maintaining access to these important services during COVID-19 is difficult [23]. The disruption in access to usual care due to pandemic related restrictions is also a considerable challenge for mental health care delivery to older adults [31, 61, 143].

Finally, addressing broader risk factors and social determinants of health is also essential to supporting mental health and psychosocial wellbeing among at-risk populations during the pandemic and in the long term. Several papers about HCWs noted the importance of addressing broader risk factors and basic needs, including by providing sufficient PPE [33, 132] and adequate rest and nutrition [28, 26, 97]. Considerations for supporting BIPOC communities include the need to understand the intersections of mental health needs with COVID-19 related stressors and experiences of stigma, discrimination and marginalization [97]. The increased awareness of structural health inequities based on racialization that is occurring during the COVID-19 pandemic is a potential catalyst for targeted efforts to address the social and structural determinants of health [141]. As evidenced by recent challenges with the outgoing US administration in 2020 [145], there is also a need for anti-racist messaging, including from national leaders, to combat race-based discrimination and stigma [153].

### Considerations for e-mental health care and psychosocial support

E-mental health options are identified as having the potential to increase access to much-needed mental health and psychosocial support among a number of at-risk populations in the context of the COVID-19 pandemic. Recommendations for people who are incarcerated include access to apps and telephone psychological support [65]. E-mental health is also recommended as a safe and effective means of providing mental health support during pregnancy [136, 51], including by engaging peer support workers who have previously experienced perinatal depression [136]. Many international migrant workers have access to smartphones, creating an opportunity to deliver online mental health supports. Liem et al. [91] call for a coordinated response to provide mental health support in a variety of languages and via multiple communication channels to migrant workers during the pandemic. There is also a need to provide enhanced, individualized supports for people with disabilities and chronic conditions during the pandemic [87]. E-mental health options can offer support to people who are in isolation and to those at higher risk of COVID-19 complications [39], but must be delivered using technologies that ensure accessibility [87, 96].

Although e-mental health approaches can be beneficial, a number of challenges and considerations are identified in the literature. The availability and quality of e-mental health care is variable across the region. Several papers recommend improving the availability of quality digital mental health supports for HCWs including via self-management and CBT-based programs delivered via apps and online platforms [86, 124, 132]. Some progress has been made in this respect, including in Wuhan, China, where digital and tele-health interventions were rapidly deployed for HCWs, including online mental health courses and hotlines to provide psychological support [27, 38]. More research evidence on the effectiveness of e-mental health interventions for children and youth is needed, including online trauma-informed psychotherapy [58], virtual CBT for sleep [74] and mental health apps specifically for adolescents [95]. One editorial noted high levels of willingness among students in Hong Kong to use online mental health supports, leading the authors to call for online counseling to be included in student support services [116].

Potential delivery-side barriers must also be considered. The need for training for mental health care providers to deliver e-mental health care is emphasized in the literature [6, 52, 60]. A number of barriers to the implementation of e-mental health care are also described, including licencing regulations limiting the geographic scope of e-mental health care delivery and payment for e-mental health care by insurance plans in the US [121]. Some providers also lack the infrastructure necessary to provide e-mental health care [29]. Infrastructure support might be especially necessary for community-based or publicly funded services that might lack resources and technical expertise [55].

Barriers to access and uptake to e-mental health care are also identified; people with existing MNS disorders, for example, may have preference for in-person options [29, 59, 31] and perceptions that e-mental health is not as effective or safe as in-person care [46]. For some types of treatment, including early psychosis intervention [60] and for patients in inpatient treatment facilities [89], hybrid models, where e-mental health is combined with in-person care, might be more appropriate. For some older adults such as people living with dementia [101], e-mental health options might be particularly challenging. A variety of options to meet the specific needs of at-risk groups, including face-to-face care when safety allows, should therefore be considered [101, 121].

Access to devices such as smartphones and high-speed Internet and technological literacy are also considerable challenges for some populations [6, 29, 75] including vulnerable children and youth [25, 69, 104, 58], older people [29] and with MNS disorders [32], people living in rural communities, in poverty [39, 56, 56], among the most vulnerable PLWH [88], and among people experiencing homelessness [91]. These barriers for people with disabilities or chronic illness and their caregivers [96], suggest that flexibility in delivery options are required [108]. For example, telephone options such as crisis hotlines may be more appropriate when offered at no cost to older adults [110]. E-mental health care may also not be accessible due to language barriers among ethnic minority and Indigenous communities [111], subpopulations of PLWH, such as ethnic and racial minorities, immigrants, and sex workers [130], diverse HCWs [134], and migrants [143], for example.

Privacy and safety concerns also emerged as an important consideration. While there are calls for increased digital and e-mental health technology supports for people experiencing domestic or intimate partner violence, identified barriers include concerns about privacy and safety if online access is monitored by the abuser [35, 50] and lack of digital or Internet access, particularly in LMICs where the digital divide by gender is higher. E-supports that include interface-level safety protocols including passwords, emergency exit buttons and detection of privacy violations based on behavioural or keystroke cues are essential [50]. Privacy concerns were also highlighted for people with MNS disorders [29, 121]. For HCWs, ensuring private space is available for them to use computers or smartphones at work was important [130]. LGBTQ+ youth expressed reluctance to use online video counselling from home due to privacy concerns but were enthusiastic about using text-based mental health supports [53].

## Discussion

Recognizing the unprecedented mental health impact of the COVID-19 pandemic and its expected disproportionate impact on at-risk populations, this review has three objectives: 1) to identify priority populations in the APEC region that are at higher risk of the negative mental health impacts of COVID-19, 2) to understand needs and gaps in access to standard and e-mental health care among these populations, and 3) to explore the potential of e-mental health to address these needs in vulnerable populations. This review also responds to a gap in literature related to equity consideration for e-mental health care in the context of the pandemic and beyond. Though the mental health impacts of emergencies such as natural disasters and conflict have been well-established [154, 155], the mental health implications of a pandemic with the breadth and impact of COVID-19 are unprecedented and still emerging [77]. The results of this review can help to inform policy and practice for targeted and equitable delivery of standard and e-mental health care to priority at-risk populations in the Asia-Pacific region and opportunities for enhanced research on equity and mental care in the context of the COVID-19 pandemic.

Based on this review, a number of gaps in the literature are evident. The identified primary research studies are predominantly cross-sectional with no randomized trials or intervention studies included. Primary research studies were also lacking for several at-risk groups, which demonstrates a gap in primary research for these vulnerable populations at the time of review. Finally, of the 21 APEC member economies, only 11 were represented in the literature, with most studies originating from China (*n* = 43) and the US (*n* = 35). Of the eight APEC members classified by the World Bank as LMICs, only two (the Philippines and Mexico) were represented. LMICs often face specific challenges that may exacerbate mental health vulnerabilities and may also contribute to added barriers to care access, including limited health and mental health system resources and capacity [129] and limited Internet availability and connectivity, especially in rural or remote areas [50]. LMICs, as highlighted by the ongoing crisis in India at the time of writing, are also expected to experience challenges and delays with vaccine acquisition and rollout [156], meaning the effects of the pandemic are likely to be prolonged. A February 2021 review of the mental health implications of COVID-19 in LMICs notes that despite 83% of the world’s population residing in LMICs, literature on the mental health impacts of the pandemic remains predominantly focused on high income countries [157]. The authors also note that despite limited research to date promising mental health policies and interventions have emerged from LMICs during the pandemic. This indicates that other countries, including high-income countries, can learn from these experiences, challenging the inequitable notion of one-directional knowledge transfer between high income and LMICs [157]. These findings confirm that there is a need for primary research across the spectrum of at-risk populations and from more APEC members, including LMICs.

Despite these gaps, several common themes emerge. While the stresses, restrictions, economic problems, and isolation imposed by the pandemic are expected to adversely affect mental health generally, at-risk populations are likely to be disproportionately affected compared to the general population. These at-risk groups share vulnerability factors including stigma, structural and racial discrimination, marginalization, and poor access to services. The necessity of enhanced government and institutional support for at-risk groups is evident. As suggested in recent literature related to COVID-19 and health research globally, collaborative consultation with specific at-risk group, including through meaningful engagement in the design of programs and services, will be necessary to ensure that mental health care and psychosocial support will be relevant and accessible [157–158] .

The considerations for e-mental health are similar to those raised for general populations but are intensified for these at-risk groups. E-mental health care approaches have previously been demonstrated to be effective, with the potential to improve access and reach to mental health care in high, middle and low-income settings [9]. There is considerable potential for e-mental health to provide more accessible and relevant care to many at-risk populations, especially in the context of COVID-19. There are also considerable challenges related to e-mental health delivery among at-risk populations. The digital divide, or the gap in access to digital technologies and infrastructure, may be greater in at-risk groups [12], resulting in further health care marginalization of the most vulnerable populations [12, 12]. Though there is limited existing literature specific to equity concerns in e-health, earlier contributions published during the pandemic also speak to the risks that the increased use of e-health technologies will exacerbate inequities in care access among marginalized populations [13, 58, 75]. Crawford et al. [159] advance a Digital Health Equity Framework that captures the interconnections of socio-economic and cultural contexts, intermediate health factors (e.g. psychosocial stressors, coping, health beliefs and behaviours), digital determinants of health, health systems as a social determinant of health and resourcing and care quality as they influence digital health equity. They note that this framework can help to guide implementation and related metrics to promote digital health equity during the pandemic and beyond.

The urgency of addressing privacy and safety barriers for people living in challenging or unsafe environments, such as LGBTQ+ youth at home with unsupportive families during lockdown [53] and people experiencing domestic violence whose abusers might monitor their use of devices or web searches [50] also emerged from this review. The challenge of risk assessment in relation to the delivery of e-mental health care is particularly urgent given the substantial vulnerabilities resulting from policies such as stay-at-home orders and physical distancing. While issues related to data privacy, security and ethics are discussed in the literature in the context of the shift to e-mental health care [160], research outlining strategies to promote safe access to e-mental health care in precarious home settings is limited and warrants more attention.

The results of this review thus suggest that e-mental health is not a ‘silver bullet’, that equity must be carefully considered, and that there remains a need for flexibility and adaptability in mental health care to ensure that the mode of delivery is appropriate, acceptable and accessible to the end user. Hybrid models of care with a combination of standard and e-mental health options are particularly recommended to enhance access to care for at-risk groups [159] . ‘Low tech’ options, such as text-based or telephone interventions, can be beneficial where privacy and safety are of concern [35, 53] and/or when there are digital literacy challenges. As recommended in relation to promoting equitable access to e-health in general [13, 159], e-mental health care must be offered in a variety of languages and be culturally validated to ensure that it is both appropriate and accessible to diverse populations such as migrant, Indigenous communities and racial or ethnic minorities [56, 12, 13, 13]. It is also essential to develop clear policies and guidelines for the delivery of e-mental health care with a focus on equity and accessibility for a variety of at-risk and vulnerable populations [73, 91]. As with standard care, it is also imperative to engage service users and people with lived experience from these at-risk groups in policy development and planning for e-mental health programs [122]. Training and capacity building for providers will also be needed to ensure effective implementation of evidence-based e-mental health care across the APEC region.

From a policy perspective, COVID-19 is harshly illuminating the need to commit to addressing the social, structural and systemic inequalities highlighted by the COVID-19 pandemic. The pandemic and its mental health impacts in the APEC region have called attention to the specific vulnerabilities faced by many priority at-risk populations. In almost all cases, this in turn demonstrates that existing marginalization and social inequities are being exacerbated during the pandemic. As Moreno et al. argue [161], this a considerable challenge that also presents an opportunity to mobilize resources to address population mental health in a way that is equitable, ensuring access to care by those most in need, and addresses many underlying risk factors contributing to mental illness and distress. In LMIC contexts, where mental health systems are often limited, the pandemic represents a chance to ‘build back better’ and to expand access to appropriate psychosocial care, including via the use of e-mental health approaches [157]. In addition to the need for more accessible mental health care for priority at-risk populations as described above, there is also a need to address the long-standing social and structural determinants of health that contribute to marginalization and poor mental health [26, 162]. Others have called attention to the critical need to address social inequalities as part of the mental health response to COVID-19 [162], and government policies and mental health responses to the pandemic must commit to addressing these entrenched inequities.

### Limitations

This review is limited to literature from the first 8 months of the pandemic, and hence represents information on early pandemic mental health needs and responses. Though this review does not capture the most recent of the rapidly emerging body of literature on the mental health impacts of the COVID-19 pandemic, it presents a very comprehensive overview of equity challenges and considerations for standard and e-mental health care that we believe will remain highly relevant for the remainder of the pandemic and beyond. As new research, including effectiveness and implementation studies, is rapidly taking place, further research is warranted to capture these emerging findings.

We have aimed to capture perspectives from across a diverse region of 21 member economies. The limitations of geographical representation in the literature at the time of review are described above. Additionally, due to resource limitations, we have only reviewed English language papers. This likely leads to the exclusion of literature in other APEC languages, however we believe that given the volume of records included in this review we have captured, to the best of our ability, a representative picture of the priorities and challenges faced in the region.

## Conclusions

The COVID-19 pandemic will have profound and long-lasting mental health impact, which will disproportionately affect at-risk populations who are often already marginalized. Given the rapid shift to the use of e-mental health care because of pandemic-related restrictions, access to digital care must be prioritized for at-risk populations to promote equity in access to care. Existing research on e-health and e-mental health equity is limited [14]. The COVID-19 pandemic, however, has underscored the urgency of addressing disparities in access to care for both standard and e-mental health care. It also is leading to increased calls for attention to equity issues in the field of digital health [12–14, 159]. This review and the associated TEAM-CAP study will lead to improved evidence and recommendations for the equitable delivery of e-mental health care across the APEC region.

Though mental health and equity challenges are experienced globally, focusing on the APEC region, which makes up 40% of the world’s population [163], represents a considerable opportunity for to inform policy and practice for equitable mental health care delivery in the context of COVID-19 and beyond. The size and diversity of the APEC region means that findings from this review can inform mental health care delivery in what is essentially a global archipelago of contexts. All countries must consider the needs of their most vulnerable populations in relation to their national priorities. The global paradigm shift resulting from the COVID-19 pandemic offers an opportunity for the APEC community to lead the way - to develop policies and programs that address inequities in mental health care access, harness the full potential of e-mental health technologies, and to address social and structural determinants of health that contribute to entrenched inequities in mental health care access.

## Data Availability

Data are available upon reasonable request from the corresponding author.

## Appendix A

### Rapid Scoping Review Database Search Terms

1. (exp coronavirus/ or coronavirus*.mp.) and (wuhan or beijing or shanghai or 2019-nCoV or nCov or COVID-19 or SARS-CoV-2).mp.

2. coronavirus*.ti. or (novel coronavirus*.mp. and (exp china/ or china.mp.)) or ((pneumonia.mp. or exp. pneumonia/) and Wuhan.mp.)

3. (‘COVID-19’ or ‘2019-nCov’ or ‘SARS-CoV-2’).Mp. Or exp. coronavirus infections/

4. or/1–3.

5. Mental health/

6. mental health.tw.

7. “psychosocial”.tw.

8. “mental illness”.tw.

9. or/5–8.

10. Vulnerable Populations/.

11. (vulnerable and (people or population*)).tw.

12. (disadvantaged and (people or population*)).tw.

13. ((homeless and (people or population*)) or “homeless*” or “at risk”).tw.

14. exp. Homeless Persons/.

15. Exp. personnel, hospital/

16. exp. Medical Staff/.

17. (“medical staff” or “nurse*” or “doctor*” or “health care provider*” or “care provider*” or “physician*” or “occupational therapist” or “physiotherapist*” or “respiratory therapist*” or “therapist*”).Tw

18. exp. Ethnic Groups/.

19. (“asian” or “black” or “hispanic” or “latino*” or “african american*” or “asian american*” or “racialized people” or “racialized group” or “minority group” or “ethnic group*” or “first nation*” or “native people*”).Tw

20. exp. “Emigrants and Immigrants”/.

21. (“emigrant*” or “immigrant*” or “refugee*”).Tw

22. exp. “Transients and Migrants”/.

23. (“transient*” or “migrant*”).tw.

24. Working Poor/.

25. (“low-SES” or “low socioeconomic status” or “poverty” or “working poor” or “low-income people” or “low-income person*”).tw.

26. Sex Workers/.

27. “sex worker*”.tw.

28. exp. Women/.

29. “Women”.Tw

30. exp. Disabled Persons/.

31. (“person with disabilities” or “person with disability” or “person with intellectual disability*” or “person with physical disability*”).tw.

32. Homebound Persons/.

33. “homebound person*”.tw.

34. Caregivers/.

35. “caregiver*”.tw.

36. exp. Aged/.

37. (“elderly” or “older adult*” or “aging population*”).Tw

38. exp. Domestic Violence/.

39. (“domestic violence” or “abusive families” or “abusive partner”).Tw

40. exp. Intimate Partner Violence/.

41. “abusive spouse”.tw.

42. Adolescent/.

43. (“adolescent” or “adolescence”).tw.

44. (“youth” or “juvenile” or “teenager*” or “student*”).tw.

45. exp. Child/.

46. “Children”.Tw

47. exp. HIV/.

48. (“people with HIV” or “person with HIV” or “person with HIV/AIDS” or “person with AIDS”).tw.

49. Minority Groups/.

50. (“sexual minority” or “racial minority” or “ethnic minority” or “people of color” or “LGBTQIA” or “gay” or “lesbian” or “transgender” or “men who have sex with men” or “LGBT*”).tw.

51. Prisoners/.

52. (“prisoner*” or “inmate*”).tw.

53. or/10–52.

54. (“Asia Pacific” or “Asia Pacific Region” or “Australia” or “Brunei Darussalam” or “Canada” or “Chile” or “People’s Republic of China” or “Hong Kong” or “China” or “Indonesia” or “Japan” or “Republic of Korea” or “Korea” or “Malaysia” or “Mexico” or “New Zealand” or “Papua New Guinea” or “Peru” or “The Philippines” or “Russia” or “Singapore” or “Chinese Taipei” or “Thailand” or “The United States” or “United States” or “Viet Nam” or “Vietnam” or “North America”).tw.

55. 4 and 9 and 53 and 54.

Publication date limit = 01-Jan-2020 to 31-July-2020.

## Abbreviations

APEC: Asia Pacific Economic Cooperation
ART: Antiretroviral Therapy
BIPOC: Black, Indigenous and People of Colour
CBT: Cognitive behavioural therapy
COVID-19: Coronavirus disease 2019
HCW: Health care worker
HIV: Human Immunodeficiency Virus
IDD: Intellectual and developmental disability
LGBTQ+: Lesbian, Gay, Bisexual, Transgender, Queer or Questioning, Plus
LMIC: Low- and middle-income country
MDD: Major Depressive Disorder
MNS: Mental, neurological and substance use
MSM: Men who have sex with men
NCD: Non-communicable disease
OCD: Obsessive-compulsive disorder
PLWH: People living with HIV
PPE: Personal protective equipment
PTSD: Post-traumatic stress disorder
PTSS: Post-traumatic stress syndrome
US: United States
SARS: Severe Acute Respiratory Syndrome
